# A broad-spectrum cloning vector that exists as both an integrated element and a free plasmid in *Chlamydia trachomatis*

**DOI:** 10.1371/journal.pone.0261088

**Published:** 2021-12-16

**Authors:** Lotisha Garvin, Rebecca Vande Voorde, Mary Dickinson, Steven Carrell, Kevin Hybiske, Daniel Rockey

**Affiliations:** 1 Department of Biomedical Sciences, Carlson College of Veterinary Medicine, Oregon State University, Corvallis, OR, United States of America; 2 Division of Allergy and Infectious Diseases, Department of Medicine, Center for Emerging and Reemerging Infectious Disease (CERID), University of Washington, Seattle, WA, United States of America; University of Texas Health Science Center at San Antonio, UNITED STATES

## Abstract

Plasmid transformation of chlamydiae has created new opportunities to investigate host–microbe interactions during chlamydial infections; however, there are still limitations. Plasmid transformation requires a replicon derived from the native *Chlamydia* plasmid, and these transformations are species-specific. We explored the utility of a broad host-range plasmid, pBBR1MCS-4, to transform chlamydiae, with a goal of simplifying the transformation process. The plasmid was modified to contain chromosomal DNA from *C*. *trachomatis* to facilitate homologous recombination. Sequences flanking *incA* were cloned into the pBBR1MCS-4 vector along with the GFP:CAT cassette from the pSW2-GFP chlamydial shuttle vector. The final plasmid construct, pBVR2, was successfully transformed into *C*. *trachomatis* strain L2-434. Chlamydial transformants were analyzed by immunofluorescence microscopy and positive clones were sequentially purified using limiting dilution. PCR and PacBio-based whole genome sequencing were used to determine if the plasmid was maintained within the chromosome or as an episome. PacBio sequencing of the cloned transformants revealed allelic exchange events between the chromosome and plasmid pBVR2 that replaced chromosomal *incA* with the plasmid GFP:CAT cassette. The data also showed evidence of full integration of the plasmid into the bacterial chromosome. While some plasmids were fully integrated, some were maintained as episomes and could be purified and retransformed into *E*. *coli*. Thus, the plasmid can be successfully transformed into chlamydia without a chlamydial origin of replication and can exist in multiple states within a transformed population.

## Introduction

*Chlamydia trachomatis* is the causative agent of the most common reportable bacterial sexually transmitted infection in the US and the leading cause of infectious blindness worldwide [1,2]. Genetic manipulation of *Chlamydia* has been challenging due to the intracellular nature of the bacteria, as well as their unique developmental cycle. The extracellular elementary bodies (EBs) have a cell wall composed of a tight matrix of proteins crosslinked by disulfide bonds [3], rendering the extracellular form of the bacteria intractable to early attempts at introducing foreign DNA. Once inside the cell, the bacteria occupy a pathogen-modified vesicle (the inclusion) where the replicating reticulate bodies (RB) are protected from the defenses of the host cell [4].

Early attempts at introducing DNA into *Chlamydia* for genetic manipulation using plasmid vectors were minimally successful. In 1994, Tam et al [5] transformed *C*. *trachomatis* by electroporation. The hybrid plasmid pPBW100 contained a promoter region and plasmid maintenance genes from *C*. *trachomatis* plasmid pCTE1 combined with a promotorless chloramphenicol acetyl transferase (*cat)* cassette and a kanamycin resistance gene from the *E*. *coli* plasmid. They recovered transient resistance to chloramphenicol (Cm) in the early stages of the lifecycle, but few transformants maintained resistance beyond four passages.

In 2005, Binet and Maurelli conducted a study that found specific mutations in the 16S rRNA operon of *C*. *psittaci* 6BC capable of conferring resistance to the aminoglycosides spectinomycin and kasugamycin [6]. To test whether homologous recombination could be used to mutate C*hlamydia*, they constructed a pUC plasmid containing the mutated 16S rRNA allele of *C*. *psittaci* 6BC and transformed it into the bacteria by electroporation [7]. They were able to successfully transfer antibiotic resistance to wild type *C*. *psittaci* 6BC, showing that homologous recombination as a means of mutagenesis was possible in *Chlamydia*. The resistance was stable; however, the investigators were only able to detect the transformation vector in less than 1% of recombinants [7].

In 2011, Wang et al used a calcium chloride treatment protocol to successfully transform the *Chlamydia* shuttle vector pRSGFPCAT into a plasmid-free mutant of *C*. *trachomatis* L2-434 [8]. This construct contained the eight open reading frames of the pL2 native plasmid with a red-shifted green fluorescent protein (GFP) fused to *cat*. This was the first study to demonstrate the stable maintenance of a transformed plasmid into *Chlamydia*, with bacteria expressing GFP and Cm resistance after multiple passages. Since the publishing of this important study, many investigators have used this transformation protocol and designed shuttle vectors with ORFs of the *Chlamydia* native plasmid to ensure maintenance of the vector [5,7,9,10].

Mueller et al [11] used fluorescence-reported allelic exchange mutagenesis (FRAEM) to exchange *trpA* with a green fluorescent protein (*gfp*) and a β- lactamase cassette. They used a tetracycline inducible system to control transcription of the pL2 ORF *pgp6*, which has been linked with plasmid growth and maintenance. By turning off transcription of this gene, the authors were able to terminate replication of the transformed vector and study homologous recombination in the system. Genetic technologies continue to be expanded in this system [11–17], each of which is dependent on transposition or a vector that includes the chlamydia plasmid replication machinery. Identification of an unrelated plasmid that does not include chlamydial plasmid sequence will add additional options to the genetics toolbox in this system.

In this study, we explored the use of a broad-host-range low copy plasmid, pBBR1MCS-4, as a transformation tool for *Chlamydia*. This Gram-negative plasmid, isolated from *Bordetella pertussis*, replicates in organisms such as *E*. *coli*, *Bartonella*, and *Pseudomonas* [18]. In *E*. *coli*, it is maintained at 30–40 copies per cell. The plasmid contains a multiple cloning site within *lacZ*α and a *bla* cassette that confers resistance to β-lactam antibiotics. It also has a unique origin of replication and encodes its own replication apparatus [18,19]. In the absence of sequence from the L2 native plasmid, we originally hypothesized that this construct would be unstable within the *Chlamydia* genome. Instead we observed genomic integration, allelic exchange, and sustained extrachromosomal replication and maintenance of the plasmid.

## Materials and methods

### Chlamydia strains and cell lines

Murine fibroblast McCoy cells (ATCC #CRL-1696) were cultured in DMEM with 10% FBS (ThermoFisher) at 37⁰C in 5% CO_2_. To propagate the parental strain *C*. *trachomatis* L2-434 and all transformed variants, McCoy cells were infected at an MOI of 0.5 for 48 hours. Cells were washed and incubated at 37⁰C in a 5% CO_2_ incubator for 48 hours, followed by the lysing of infected monolayers by a -80⁰C/37⁰C freeze/thaw. Cells were then harvested from flasks and centrifuged at 448 x *g* for 5 min. The supernatant was transferred to 1.5 ml tubes and centrifuged at 3000 x *g* for 15 min. The supernatant was discarded, and the pelleted bacteria were suspended in 1 ml SPG (0.25 M sucrose, 10 mM sodium phosphate, 5 mM L-glutamic acid, pH 7.2), divided into aliquots, and stored at -80⁰C for future use. For all transformation experiments McCoy cells were grown to 80% confluence in a T-75 flask. Medium was removed and the cells were washed twice with PBS. Attached cells were trypsinized, washed, and seeded at 1x10^5^ cells/well in a 6- well plate and allowed to attach overnight.

### pBVR plasmid construction

The *incG* coding sequence from *C*. *trachomatis* serovar L2-434 was PCR amplified using primers RV238 and RV239 (S1 Table) with terminal *PstI* and *XbaI* restriction enzyme sites incorporated at the 5’ and 3’ ends, respectively. Both vector pBBR1MCS-4 [19] and the PCR product were digested with *PstI* and *XbaI* restriction enzymes for 1 h at 37⁰ C. Digested DNAs were electrophoresed and purified using a GeneJET gel Extraction kit (Thermo Fisher), and ligated prior to transformation into *E*.*coli* Top10 cells (NEB). For selection of transformants, individual ampicillin (Amp)-resistant colonies were isolated from selective media. Colonies that were positive for the *incG* insert by PCR were grown overnight in LB broth with 100 μg/ml Amp, and plasmid DNA was prepared. The presence of *incG* was confirmed by restriction digest analysis. This plasmid was designated pBVR1.

### Generation of pBVR2

The *ct_120* open reading frame from *C*. *trachomatis* L2-434 was amplified from genomic DNA via primers RV240 and RV263 (S1 Table) incorporating *SalI* and *KpnI* restriction sites, respectively, on the 5’ and 3’ends. The *gfp*:*cat* cassette from the chlamydial shuttle vector pSW2-GFP was digested with *SalI* and *PstI* and the 1908 bp fragment was purified with GeneJET purification kit (Thermo Fisher). A triple ligation was then performed using *PstI*- and *KpnI*- digested pBVR1 DNA, *SalI* and *PstI*-digested *gfp*:*cat* DNA and *SalI*- and *KpnI*- digested *ct_120*. The ligation mix was used to transform *E*. *coli* Top10 (NEB) cells, and transformants were selected via resistance to Amp and Cm. Colonies were streaked for isolation and screened for the presence of *ct_120* by PCR using primers RV240 and RV263. Colonies positive for *ct_120* were grown in LB broth culture plus 100 μg/ml Amp for plasmid isolation and were sequenced using Sanger sequencing. The plasmid, once confirmed to contain all required elements, was designated pBVR2 (S1 Fig), and transformed into *E*. *coli dam*-/*dcm*- competent cells (NEB C2925I), grown in 250 ml of LB broth plus 100 μg/ml Amp for plasmid extraction and purified using a Plasmid Maxi kit (Qiagen) in preparation for transformation into *C*. *trachomatis*.

### Transformation of pBVR2 into *C*. *trachomatis* L2-434

Plasmid transformation was performed as previously described by Wang et al [9] with minor modifications. 1x10^7^ EBs in 10 μl were incubated with 3 μg of pBVR2 plasmid DNA and mixed with 100 μl of CaCl_2_ buffer (10 mM Tris pH 7.4 and 50 mM CaCl_2_). The mixture was incubated at room temperature for 30 min. After incubation, 50 μl of plasmid/EB mix and 2 ml of DMEM +10% FBS was added to a single well of a 6-well plate containing a monolayer of McCoy cells. This was incubated with shaking for 10 minutes. The 6-well plate was centrifuged at 400 x *g* for 30 min and the medium was replaced with fresh DMEM. Infected cells were incubated at 37⁰ C 5% CO_2_ for 4 hours, after which 0.5 μg/ml Cm was added for selection, and 1 μg/ml cycloheximide was added to halt host cell protein synthesis. Infections were incubated for 48 hours before the first passage.

### Passage and propagation of GFP positive transformants

Cell culture medium was removed from the monolayers and adherent cells were washed two times with PBS. Cells were then lysed by addition of 100 μl dH_2_O to each well. After a 15 min incubation at room temperature, the monolayer in each well was lifted, and the suspension was pipetted a minimum of 10 times to break up clumps. 200 μl of 4SP buffer (0.4 M sucrose, 0.04 M phosphate buffer, pH 7.2) was added to each well and thoroughly mixed. This was then added to a fresh monolayer of McCoy cells on a 6-well plate. The plate was centrifuged at 400 x *g* for 30 min, washed, and the medium replaced with DMEM containing 0.5 μg/ml Cm and 1 μg/ml cycloheximide. This was repeated every 48–72 hours until the appearance of normal inclusions containing GFP-expressing bacteria were observed by fluorescence microscopy. Transformants were selected for with 0.5 μg/ml Cm and expanded in McCoy cells for further use.

### Clonal expansion of plasmid-containing *C*. *trachomatis*

Transformants were cloned by using a modification of a limiting dilution method as previously described [20]. Briefly, candidate chlamydial transformants were serially diluted ten-fold and plated onto a 96 well plate. McCoy cells were incubated for 4–5 days and scanned for the presence of individual GFP-positive inclusions by fluorescence microscopy. Clones identified in these wells were propagated until the MOI approached 0.5. Individual wells were then passed to fresh monolayers of increasingly larger surface area until clonal populations were being propagated in T-75 flasks. The resulting transformants underwent multiple rounds of cloning and expansion in the presence of appropriate antibiotics.

### Genomic DNA and plasmid extraction from cloned transformants

Chromosomal DNA and plasmid DNA were prepared from *C*. *trachomatis* pBVR2-positive clones. Host cell DNA was first depleted using DNAse I treatment as previously described [21]. Purified EBs were centrifuged at 3000 x *g* for 15 min and the pellet incubated in a RQ1 DNase master mix (Promega) at 37°C for 30 min. DNA digestion was terminated by addition of RQ1 stop buffer and a 10-minute incubation at 65°C. DNA from treated samples was then extracted using the DNeasy Blood and Tissue kit (Qiagen) per the manufacturer’s instructions. DNA for extraction of pBVR2 plasmid from transformed *C*. *trachomatis* was processed as described above, and the GeneJet Plasmid miniprep kit (Thermo Fisher) was used for plasmid purification. The resuspension buffer was supplemented with 5 mM of dithiothreitol during the plasmid extraction protocol.

### Transformation of *E*. *coli* with plasmid extracted from *C*. *trachomatis*

Extracted chlamydial plasmid was transformed into chemically competent *E*. *coli* Top10 cells (NEB) according to manufacturer’s instructions. Briefly, 3 ng of plasmid DNA was mixed with chemically competent cells and incubated on ice for 30 min. Cells were incubated at 42⁰ C for 30 sec and placed on ice for 5 min after which 950 μl of SOC outgrowth media (NEB) were added to the mixture. Transformed bacteria were then incubated at 37⁰ C with shaking for 1 h. The culture was then centrifuged for 10 min at 16,100 x *g*, suspended in 100 μl SOC and plated on LB agar containing Amp (100 μg/ml) and Cm (25 μg/ml). Positive transformants were isolated and propagated in LB broth culture overnight for plasmid maxi-prep extraction as previously described. Purified plasmid was digested with *SalI* and *KpnI* and compared to the original pBVR2 plasmid.

### Immunofluorescence analysis of chlamydial transformants

Antibody labelling was performed on infected McCoy cell monolayers as described previously [22]. Live cells were screened for GFP expression using a Fluorescein (FITC) filter. Cells were then fixed with 100% methanol for 15 min at room temperature and incubated with 2% bovine serum albumin (BSA) in PBS. Mouse-derived monoclonal antibodies to *C*. *trachomatis* IncA [23] were added to the monolayer, followed by fluorescein conjugated secondary antibody (Southern Biotechnology Associates). DAPI (4,6- diamidino-2-phenylindole) in VectaShield mounting medium (VectaShield) was used to label bacterial DNA and host cell nuclei. Staining was visualized using a Leica LB 100T fluorescence microscope, and images were analyzed using QCapPro60 software (QImaging, Inc).

### Analysis of plasmid integration in *C*. *trachomatis* chromosome

Primer sets were designed to determine the presence of the insert sequence in relation to the genome (S1 Table). PCR amplification using DNA from the pBVR2 transformants was performed using primers specific for *C*. *trachomatis* DNA within the chromosome and outside of the cloned region (RV294 or RV295), and a primer specific for the pBVR2 insert *gfp*:*cat* (RV296 or RV311). PCR product from transformed *C*. *trachomatis* was sequenced to confirm the insertion sites.

### Whole genome sequencing

pBVR2 transformants were cultured to a high concentration (~ 1x10^7^ IFU) for 48 hours. The infected monolayer was exposed to sterile water for 10 minutes to lyse the cells. This lysate was then centrifuged at 448 x *g* for 5 min and the supernatant was centrifuged at 3000 x *g* for 15 minutes and the supernatant was discarded. The pellet was suspended in water, treated with RQ1 DNase (Promega) at 37⁰ C for 30 min, and incubated in RQ1 stop solution (10 min at 65⁰C) after which dithiothreitol was added, to a final concentration of 5 mM. After a 1 h incubation at 56⁰ C, genomic DNA was extracted using the DNeasy Blood and Tissue Kit (Qiagen). Whole genome sequencing templates were prepared using SMRTBell Express Template prep kit (Pacbio). Sequencing was performed by PacBio SMRT Sequencing at the Oregon State University Center for Genome Research and Biocomputing. PacBio-generated reads were assembled using Geneious software [24]. To determine the nature of the plasmid insertion, read data were mapped with the reference GFP marker using low sensitivity. Reads associated with GFP were further characterized by mapping sequences to the *C*. *trachomatis* L2-434 genome. Genes were annotated on the read data before alignments were generated using the Needleman-Wunsch Global aligner, at a 70% similarity cost-matrix, to determine the nature of the insertion and the insertion site. All 4 files are available from the NCBI database (accession numbers SAMN19816454, SAMN19816455, SAMN19816456, SAMN19816457). Bioproject: PRJNA740046.

## Results

### Transformation of pBVR2 plasmid into *C*. *trachomatis*

**Fig 1:**
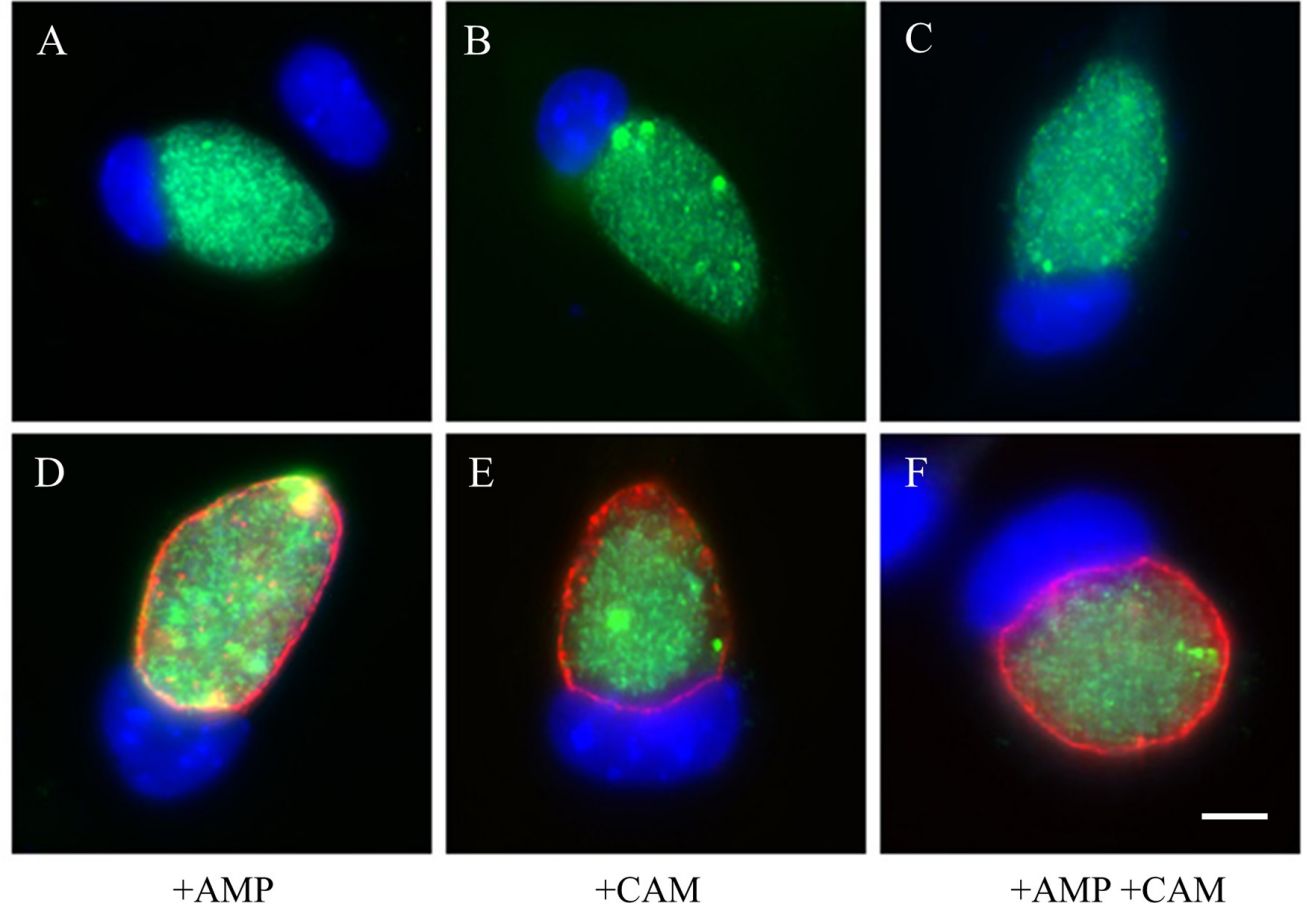
Immunofluorescence images of pBVR2 transformants show GFP and IncA expression. GFP fluorescence of chlamydial inclusions was visualized in live cells prior to fixing and staining. Infected McCoy cells were fixed with 100% MeOH at 48hpi. All cells were stained with DAPI to visualize host cell nuclei (blue). **(A-C)** Unlabeled GFP positive bacteria. **(D-F)** Cells labeled with antibodies to *C*. *trachomatis* IncA (red). **(A, D)** Cells treated with 100 μg/ml Amp only. **(B, E)** Cells treated with 1 μg/ml Cm only. **(C, F)** Cells treated with 100 μg/ml Amp and 1 μg/ml Cm. Scale bar represents 5 μm.

**Fig 2:**
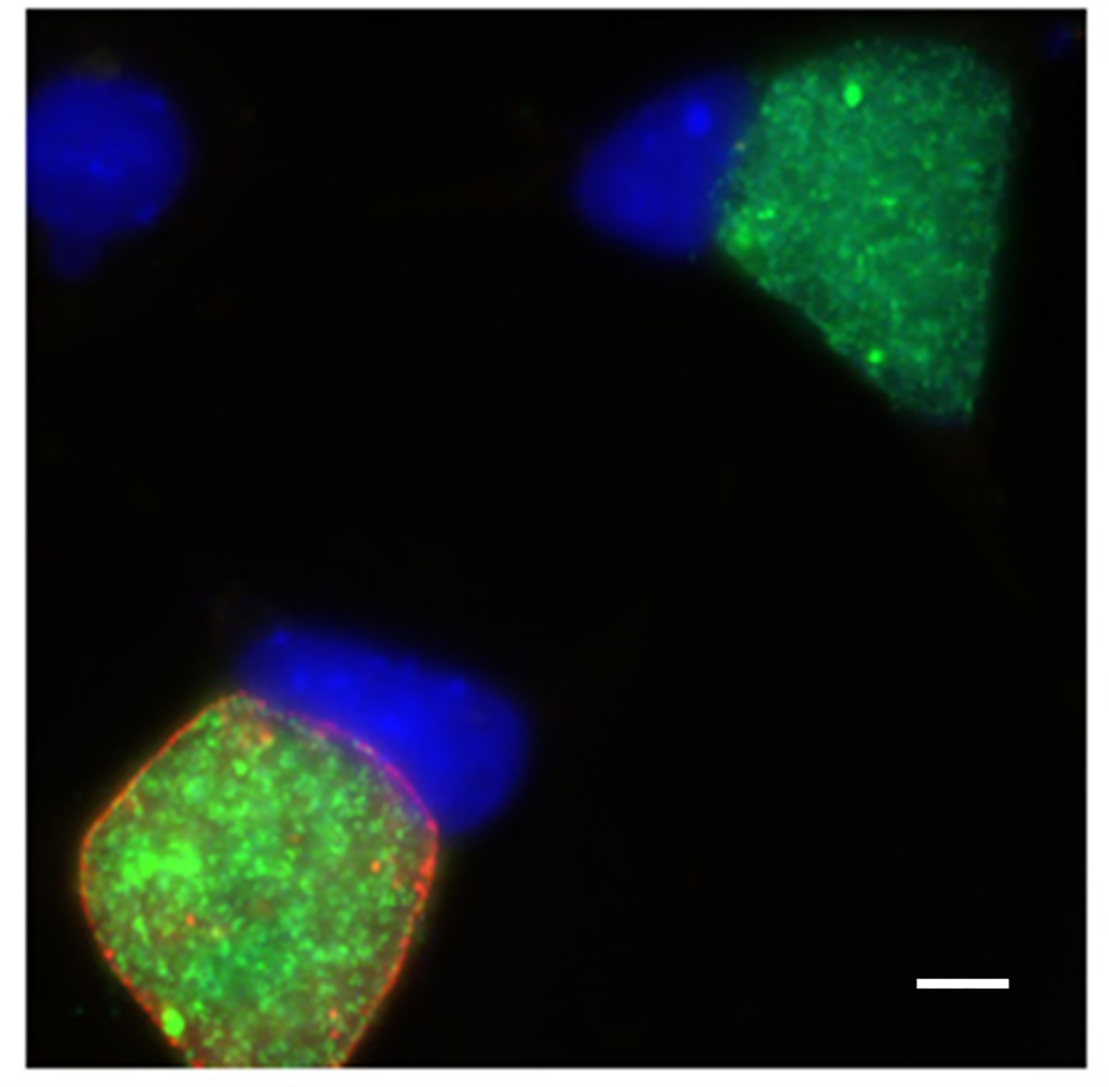
pBVR2 transformants exhibit heterogenous expression of IncA. GFP fluorescence of chlamydial inclusions was visualized in live cells prior to fixing and staining. The infected McCoy cells were fixed with 100% MeOH at 48 hpi and labeled with antibody against *C*. *trachomatis* IncA (red). The monolayer was then stained with DAPI to visualize host cell nuclei (blue). Scale bar represents 5 μm.

We first sought to determine whether we could successfully transform *C*. *trachomatis* (serovar L2) with pBVR2, a plasmid with a non-chlamydial origin of replication (S1 Fig). After 3 passages in cell culture with medium containing 1 μg/ml of Cm, we recovered GFP-positive bacteria with resistance to both Cm and Amp (Fig 1), indicating the successful recovery of pBVR2 transformants. Microscopy of the initial group of transformants showed conserved expression of IncA (Fig 1), a protein known to reside in the chlamydial inclusion membrane [22], suggesting that the expected double crossover between the chromosomal *incA* and the plasmid-sourced *gfp*:*cat* cassette had not taken place. However, transformants that had undergone >5 passages exhibited variable IncA positivity while maintaining Cm and Amp resistance in all clones (Fig 2). This suggested that homologous recombination had taken place either at the *incG* or *ct_120* loci in the chromosome and those on plasmid pBVR2. The continued resistance to Amp demonstrated that the bacteria retained the plasmid-encoded *bla* marker.

Multiple, parallel attempts to transform the parent pBBR1MCS-4 vector into *C*. *trachomatis*, by three different individuals, were not successful.

### Evidence for free pBVR2 plasmid in EBs

**Fig 3:**
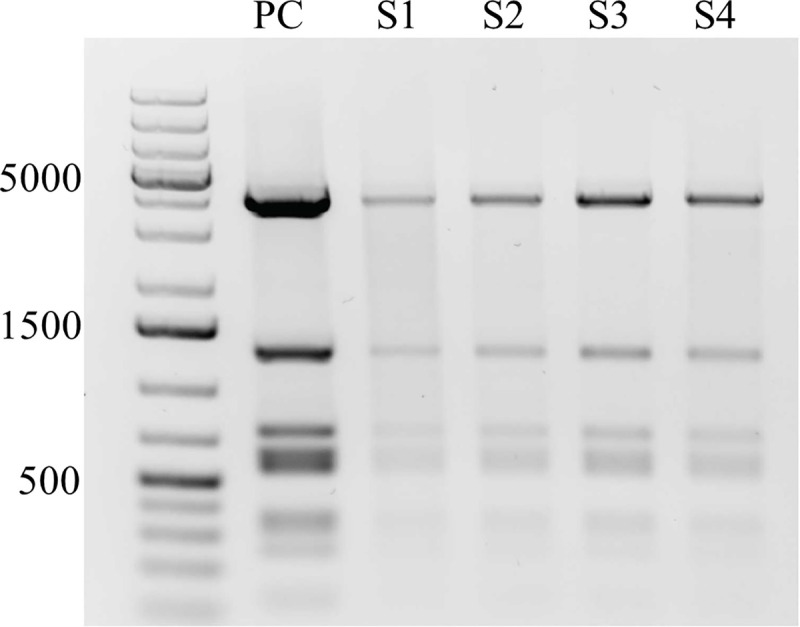
*C*. *trachomatis* derived pBVR2 plasmid is identical to *E*. *coli* derived pBVR2 plasmid. Plasmid isolated from *C*. *trachomatis* and *E*. *coli* were digested with restriction enzymes *AdeI* and *BglII*. (PC) positive control pBVR2 plasmid raised in *dam*-/*dcm*- *E*. *coli* for initial transformation of *C*. *trachomatis*. (S1-S2) clones of pBVR2 plasmid isolated during round 1 of transformation that was extracted from *C*. *trachomatis* and transformed into *E*. *coli* Top10 cells. (S3-S4) Clones were isolated during second round of cloning using same protocol as round 1 clones.

The observation that some transformants exhibited variable IncA expression while all maintained resistance to Amp led us to question if the plasmid was able to be maintained as an episome in addition to being integrated into the chromosome. To address this, plasmid purified from clonal isolates was transformed into *E*. *coli*. Restriction enzyme mapping of the purified *E*. *coli* plasmids revealed identical patterns between the original pBVR2 plasmid and the isolated clones (Fig 3). Sequencing of the amplified *incG*:*gfp*:*cat* region from transformed *C*. *trachomatis* showed that the transformed DNA was identical to the predicted 1389 bp of the original insertion. A second round of limiting dilution generated a subcloned population of chlamydial transformants. Two of these clones were isolated for plasmid purification and characterization, and the restriction mapping patterns of the plasmids were identical to the original pBVR2 plasmid. These data indicated that the pBVR2 plasmid was successfully acquired by the transformants and that the plasmid was stably maintained through multiple passages.

### pBVR2 integrates into the chromosome of *C*. *trachomatis*

**Fig 4:**
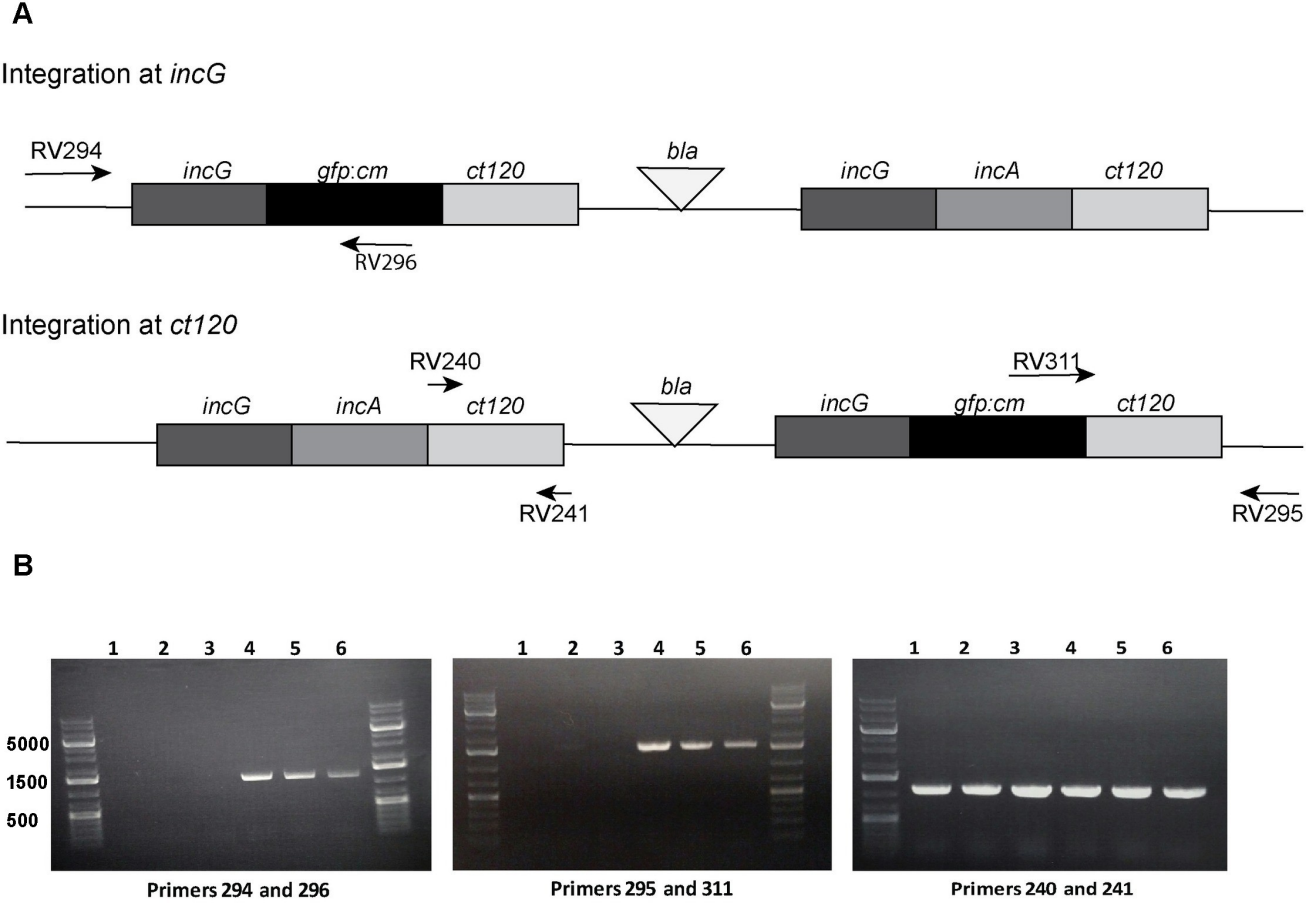
Plasmid pBVR2 integrates into the *C*. *trachomatis* chromosome at homologous regions. PCR amplification using DNA from the pBVR2 transformants was performed using primers specific for *C*. *trachomatis* chromosomal DNA: One outside of the cloned region (294 or 295), and one located within *gfp*:*cat* (296 or 311). Negative controls included non-transformed *C*. *trachomatis* L2 DNA (lane 1), *C*. *trachomatis* L2 transformed with plasmid pSW2 (lane 2), and plasmid pBVR2 DNA alone (lane 3). Primers 240 and 241, specific for *ct_120*, were used as a positive PCR control. Lanes (4–5) contained chromosomal DNA from the *C*. *trachomatis* pBVR2 clones 5F and 6B. Lane (6) contained chromosomal DNA from the parent *C*. *trachomatis* pBVR2 transformant prior to cloning.

We next examined whether the transformants were integrating into the *C*. *trachomatis* chromosome. PCR primer sets were designed to determine whether integration of the plasmid occurred at either the *incG* or *ct_120* sites of homology (Fig 4A). Primers with sequences located internally to the plasmid sequence were paired with a primer externally located within the bacterial chromosome at both the *incG* and *ct_120* sites. The presence of PCR products for both sets of primers in the isolated clones indicates that the integration of the plasmid had occurred independently within *incG* or *ct_120* coding sequences (Fig 4B). These data indicate that the plasmid was able to integrate into the chromosome at both regions of sequence identity.

### PACBIO genome sequencing

**Fig 5:**
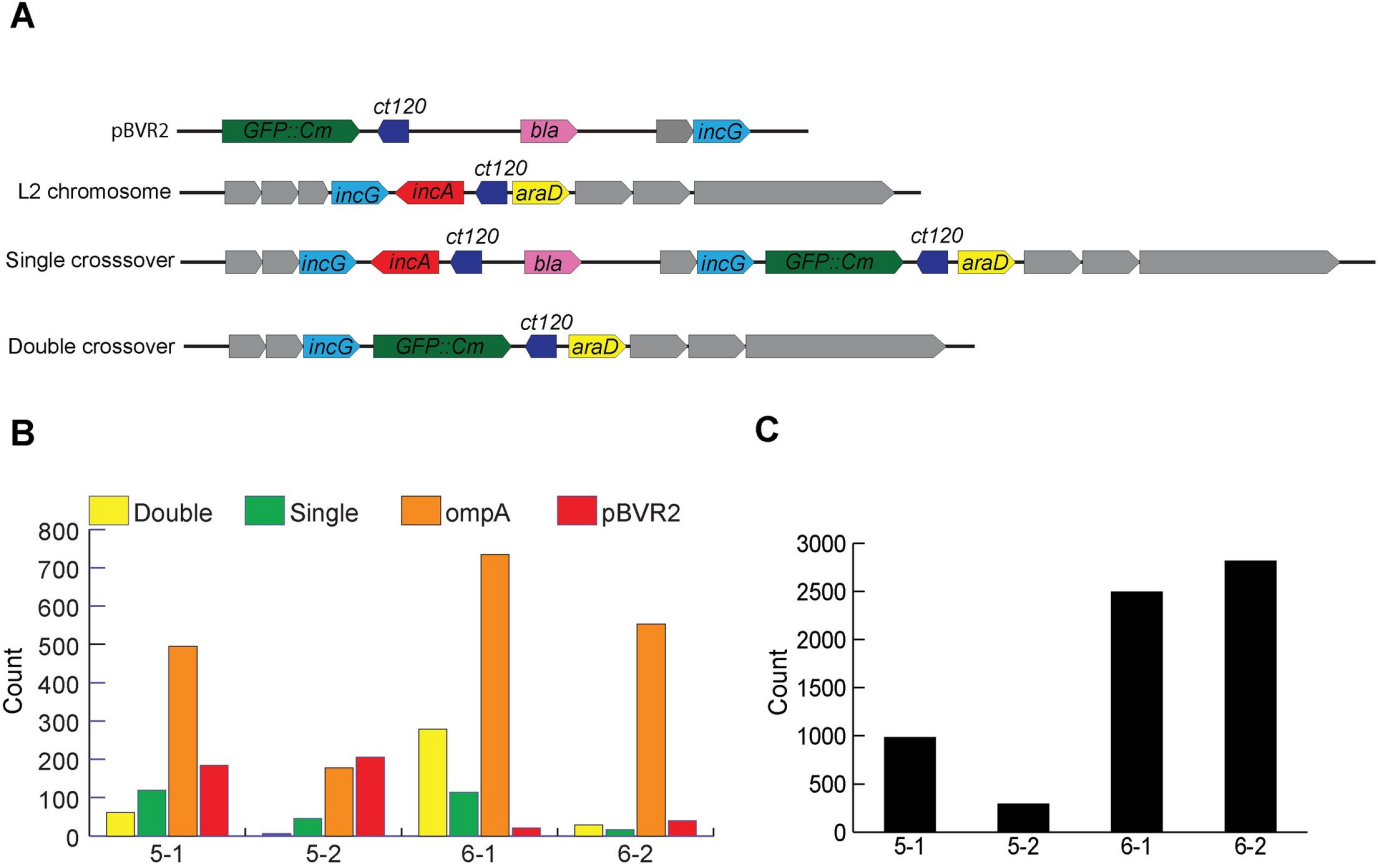
Plasmid pBVR2 exists as both an integration into the *C*. *trachomatis* chromosome and an episomal unit. *C*. *trachomatis* genomic DNA was sequenced via PACBIO whole genome sequencing, and the reads were assembled and parsed for the presence of genes indicating the integration of pBVR2 plasmid in the chromosome. (**A**) Gene map of pBVR plasmid and possible chromosome integration scenarios. (**B**) Counts of assembled whole genome read data parsed to determine existence of different integration sequences. The labels 5–1 and 5–2 denote the *C*. *trachomatis* clones derived from isolate 5B while 6–1 and 6–2 denote clones derived from isolate 6F.(**C**) Counts of assembled read data denoting presence of pL2 native plasmid. Note: pBVR2 plasmid can integrate into chromosome from either the *incG* or the *ct_120* regions of homology, though only one representative single crossover is shown in the gene map.

We then performed whole genome sequencing on the cloned *C*. *trachomatis* pBVR2 transformants to identify single or double crossover events between the plasmid and chromosome (Fig 5A). Using a PACBIO platform for collection of sequence data, we searched the assembled reads for sequences demonstrating either integrated or free plasmid. The presence of free pBVR2 plasmid in the cell was determined by first examining all reads that were long enough to contain the full plasmid sequence. The resulting reads were then analyzed for the presence of *gfp* and *ct_120*, both located on the plasmid. Reads that contained both genes were then mapped to *araD*, which is located within the chromosome, 98 bp downstream of the recombination site. Sequences that contained both *gfp* and *ct_120* but did not contain chromosomal *araD* were indicative of free plasmid. Full, free plasmid sequence was detected in 206 of 964,718 total reads for *C*. *trachomatis* L2_pBVR2_5F clones (5–1 and 5–2), and 246 out of 1,143,297 total reads for both 6B clones (6–1 and 6–2) (Fig 5B). The presence of chromosomal *incA* and plasmid GFP: *cat* cassette occurring in the same read with flanking chromosomal regions was indicative of full integration of the plasmid. This was seen in 166 out of 964,718 total reads for both 5F clones, and 130 out of 1,143,297 total reads for both 6B clones (Fig 5B). A double- crossover event was represented by the presence of the plasmid-based *gfp*:*cat* cassette and its flanking chromosomal regions, but without *incA*. This was seen in 67 of 964,718 total reads for both rounds of 5F clones, and 308 of 1,143,297 total reads for both rounds of 6B clones. Relative read counts for *ompA* and 16S rDNA were analyzed to establish a baseline copy number for the bacteria. The presence of the native plasmid pL2 was also assessed using this method. Sequences from all the analyzed samples revealed 6,586 reads that contained the full pL2 plasmid sequence (Fig 5C), indicating that the native plasmid and the pBVR2 plasmid were able to coexist as part of the bacterial genome.

## Discussion

The described experiments demonstrate the extended stability of a plasmid transformed into *C*. *trachomatis* without the use of native plasmid-maintenance ORFs. We also describe the integration and homologous recombination of the plasmid into the *C*. *trachomatis* chromosome. Maintenance of the plasmid as an episome in this system allows novel opportunities for exploration of pBVR2, and other future adaptations of the parent broad-spectrum plasmid pBBR1MCS-4, as possible tools of discovery in the chlamydial system. This may allow novel approaches that do not depend on chlamydia-plasmid-based systems [7,9,20,25]. In our study, we were able to maintain both native plasmid and our introduced vector, perhaps in part due in part to the existence of replication apparatus native to the background pBBR1 plasmid. The potential advantage of this is that the maintenance of the native chlamydial plasmid allows analysis of the bacteria in a genetic background closer to wildtype. Further work needs to be done to define the requirements for pBBR1-based plasmids to remain freely replicating within chlamydiae, and to evaluate their utility as broad-range vectors for expression analyses in chlamydiae.

The successful use of the original pBBR1MCS4 plasmid in multiple gram-negative systems was the catalyst for the decision to utilize this vector as a shuttle for our chlamydial genes. Unfortunately, our efforts to transform *C*. *trachomatis* with the parent pBBR1 were unsuccessful. We continue to explore the use of the parent vector as a tool of transformation in chlamydiae.

The PACBIO data was used to carefully examine the nature of plasmid-chromosomal interactions in this system. The sequencing data showed that allelic exchange had taken place, however the data also showed the presence of integrated and free plasmid. This corroborates the results of the fluorescence microscopy that showed variable detection of IncA. Attempts to perform limiting dilutions to isolate the IncA-negative transformants were unsuccessful and therefore the information that we have regarding the rate of allelic exchange comes exclusively from the PACBIO data.

In addition to allelic exchange, we also saw complete integration of the plasmid into the chromosome, which resulted in the presence of multiple copies of *incF*, *incG*, and CT120. Our genome sequencing on the PACBIO platform demonstrates that the plasmid exists in multiple states within the genome, as either a free entity or integrated into the chromosome at sites of homology. The exact mechanism responsible for the multiple states is unclear. We hypothesize that the initial steps following DNA uptake involved a single homologous recombination event that integrated the into the chromosome, followed by dynamic events including a second crossover that removed wild type *incA*, the excision of the plasmid from the chromosome, and the maintenance of the plasmid as an episome. Our data support this interpretation, as limiting dilution did not yield clonal population of any of these genetic states.

Given the success of transformation of several different chlamydial species with endogenous plasmid [7,9,25,26], it is unlikely that introduction of the DNA into chlamydia is the limiting factor. Recently Shima et al transformed a pRSGFPCAT *C*. *pneumoniae* derivative into both a human and a koala strain of *C*. *pneumoniae* as well as a strain of *C*. *felis* [25], showing that cross species transformation with endogenous plasmid is possible. This gives strength to our claims that we can use our pBVR construct for this purpose. However, in the Shima study they were unable to transform this plasmid into any other species of chlamydiae tested, including *C*. *trachomatis*, *C*. *muridarum*, or *C*. *abortus*. Unlike the pRSGFPCAT vector used in that study, pBBR1MCS-4 encodes its own replication factors, and thus, we anticipate that success in one species would lead to success with other species.

Information regarding chlamydial intra-species recombination and gene transfer is lacking. We know from examination of clinical *C*. *trachomatis* isolates that recombination within species occurs readily during natural infection [27,28], and can also be achieved in a laboratory setting [29–31]. Recent studies done by our group have shown that genetic recombination is possible between Chlamydial species [31,32] and those studies are ongoing. To study the phenomenon of intra-species gene transfer would require the use of non-*C*. *trachomatis* species to explore species pathogenic specificity and host tropism.

In this work, we have identified a simple technique to generate transformants and recombinants within chlamydiae. The versatility of pBBR1-MCS4-based vectors could enable us to develop inducible systems, such as those designed to deliver counter-selectable markers to recombinant strains. The ability to express genes in *trans* without the need to construct novel specific plasmids for each species would genuinely revolutionize chlamydial genetics. Further progress in this area may significantly contribute to the repertoire of genetic tools that are currently available for chlamydia and could greatly facilitate studies of intra- or cross species complementation studies in this organism.

## Supporting information

S1 Fig The CDSs of *bla* and *lacZ* of the original pBBR1 plasmid are shown in blue and red, respectively. The green fluorescent protein fused with *cat* is shown in black.
(TIF)

S1 Table Primers used in the study. (PDF)

S1 Raw data Raw gel data for Garvin et al., Figs 3 and 4. (PDF)

## References

[1] World Health Organization. Trachoma 2020 [23 Dec 2020]. Available from: https://www.who.int/news-room/fact-sheets/detail/trachoma.

[2] World Health Organization. Report on global sexually transmitted infection surveillance 2018. Geneva: World Health Organization, 2018.

[3] Nelson DE. The Chlamydial Cell Envelope. In: Tan M, Bavoil PM, Tan M, Bavoil PM, editors. Intracellular pathogens I Chlamydiales. Washington, D.C.: Washington, D.C.: ASM Press; 2012.

[4] Elwell C, Mirrashidi K, Engel J. Chlamydia cell biology and pathogenesis. Nature reviews Microbiology. 2016;14(6):385–400. Epub 2016/04/26. doi: 10.1038/nrmicro.2016.30 ; PubMed Central PMCID: PMC4886739. 27108705 PMC4886739

[5] Tam JE, Davis CH, Wyrick PB. Expression of recombinant DNA introduced into *Chlamydia trachomatis* by electroporation. Canadian Journal of Microbiology. 1994;40(7):583–91. Epub 1994/07/01. doi: 10.1139/m94-093 8076253

[6] Binet R, Maurelli AT. Frequency of spontaneous mutations that confer antibiotic resistance in *Chlamydia* spp. Antimicrobial Agents and Chemotherapy. 2005;49(7):2865. doi: 10.1128/AAC.49.7.2865-2873.2005 15980362 PMC1168699

[7] Binet R, Maurelli AT. Transformation and isolation of allelic exchange mutants of *Chlamydia psittaci* using recombinant DNA introduced by electroporation. Proc Natl Acad Sci U S A. 2009;106(1):292–7. Epub 2008/12/24. doi: 10.1073/pnas.0806768106 ; PubMed Central PMCID: PMC2629194. 19104068 PMC2629194

[8] Wang J, Frohlich KM, Buckner L, Quayle AJ, Luo M, Feng X, et al. Altered protein secretion of *Chlamydia trachomatis* in persistently infected human endocervical epithelial cells. Microbiology. 2011;157(Pt 10):2759–71. Epub 2011/07/09. doi: 10.1099/mic.0.044917-0 ; PubMed Central PMCID: PMC3353392. 21737500 PMC3353392

[9] Wang Y, Kahane S, Cutcliffe LT, Skilton RJ, Lambden PR, Clarke IN. Development of a transformation system for *Chlamydia trachomatis*: restoration of glycogen biosynthesis by acquisition of a plasmid shuttle vector. PLoS Pathog. 2011;7(9):e1002258. Epub 2011/10/04. doi: 10.1371/journal.ppat.1002258 ; PubMed Central PMCID: PMC3178582. 21966270 PMC3178582

[10] Davis CH, Tam J.E., and Wyrick P.B. Development of a shuttle vector for *Chlamydia trachomatis*. In: William R. Bowie HDC, Jones Robert P., Mardh Per-Anders, Ridgway Geoff L., Schachter Julius, Stamm Walter E., Ward Michael E., editor. Chlamydial Infections. New York, NY: Cambridge University Press; 1990. p. 149–52.

[11] Mueller KE, Wolf K, Fields KA. Gene deletion by fluorescence-reported allelic exchange mutagenesis in *Chlamydia trachomatis*. MBio. 2016;7(1):e01817–15. Epub 2016/01/21. doi: 10.1128/mBio.01817-15 ; PubMed Central PMCID: PMC4725004. 26787828 PMC4725004

[12] Weber MM, Faris R. Mutagenesis of *Chlamydia trachomatis* using TargeTron. Methods in Molecular Biology (Clifton, NJ). 2019;2042:165–84. Epub 2019/08/07. doi: 10.1007/978-1-4939-9694-0_12 . 31385276

[13] Filcek K, Vielfort K, Muraleedharan S, Henriksson J, Valdivia RH, Bavoil PM, et al. Insertional mutagenesis in the zoonotic pathogen *Chlamydia caviae*. PLoS One. 2019;14(11):e0224324. Epub 2019/11/08. doi: 10.1371/journal.pone.0224324 ; PubMed Central PMCID: PMC6837515. 31697687 PMC6837515

[14] Johnson CM, Fisher DJ. Site-specific, insertional inactivation of incA in *Chlamydia trachomatis* using a group II intron. PLoS One. 2013;8(12):e83989. Epub 2014/01/07. doi: 10.1371/journal.pone.0083989 ; PubMed Central PMCID: PMC3877132. 24391860 PMC3877132

[15] Kari L, Goheen MM, Randall LB, Taylor LD, Carlson JH, Whitmire WM, et al. Generation of targeted *Chlamydia trachomatis* null mutants. Proceedings of the National Academy of Sciences. 2011;108(17):7189–93. doi: 10.1073/pnas.1102229108 21482792 PMC3084044

[16] Keb G, Hayman R, Fields KA. Floxed-cassette allelic exchange mutagenesis enables markerless gene deletion in *Chlamydia trachomatis* and can reverse cassette-induced polar effects. J Bacteriol. 2018;200(24). Epub 2018/09/19. doi: 10.1128/JB.00479-18 ; PubMed Central PMCID: PMC6256029. 30224436 PMC6256029

[17] McKuen MJ, Mueller KE, Bae YS, Fields KA. Fluorescence-reported allelic exchange mutagenesis reveals a role for *Chlamydia trachomatis* TmeA in invasion that is independent of host AHNAK. Infection and Immunity. 2017;85(12):e00640–17. doi: 10.1128/IAI.00640-17 . 28970272 PMC5695130

[18] Antoine R, Locht C. Isolation and molecular characterization of a novel broad-host-range plasmid from *Bordetella bronchiseptica* with sequence similarities to plasmids from Gram-positive organisms. Molecular Microbiology. 1992;6(13):1785–99. doi: 10.1111/j.1365-2958.1992.tb01351.x 1321324

[19] Kovach ME, Elzer PH, Hill DS, Robertson GT, Farris MA, Roop RM 2nd, et al. Four new derivatives of the broad-host-range cloning vector pBBR1MCS, carrying different antibiotic-resistance cassettes. Gene. 1995;166(1):175–6. Epub 1995/12/01. doi: 10.1016/0378-1119(95)00584-1 . 8529885

[20] Mueller KE, Wolf K, Fields KA. *Chlamydia trachomatis* transformation and allelic exchange mutagenesis. Current Protocols in Microbiology. 2017;45:11a.3.1–a.3.5. Epub 2017/05/17. doi: 10.1002/cpmc.31 ; PubMed Central PMCID: PMC5545879. 28510361 PMC5545879

[21] Putman TE, Suchland RJ, Ivanovitch JD, Rockey DD. Culture-independent sequence analysis of *Chlamydia trachomatis* in urogenital specimens identifies regions of recombination and in-patient sequence mutations. Microbiology (Reading, England). 2013;159(Pt 10):2109–17. doi: 10.1099/mic.0.070029–0 . 23842467 PMC3799229

[22] Bannantine JP, Griffiths RS, Viratyosin W, Brown WJ, Rockey DD. A secondary structure motif predictive of protein localization to the chlamydial inclusion membrane. Cellular Microbiology. 2000;2(1):35–47. Epub 2001/02/24. doi: 10.1046/j.1462-5822.2000.00029.x . 11207561

[23] Bannantine JP, Stamm WE, Suchland RJ, Rockey DD. *Chlamydia trachomatis* IncA is localized to the inclusion membrane and is recognized by antisera from infected humans and primates. Infect Immun. 1998;66(12):6017–21. Epub 1998/11/24. doi: 10.1128/IAI.66.12.6017-6021.1998 ; PubMed Central PMCID: PMC108764. 9826388 PMC108764

[24] Kearse M, Moir R, Wilson A, Stones-Havas S, Cheung M, Sturrock S, et al. Geneious Basic: an integrated and extendable desktop software platform for the organization and analysis of sequence data. Bioinformatics (Oxford, England). 2012;28(12):1647–9. Epub 2012/05/01. doi: 10.1093/bioinformatics/bts199 ; PubMed Central PMCID: PMC3371832. 22543367 PMC3371832

[25] Shima K, Wanker M, Skilton RJ, Cutcliffe LT, Schnee C, Kohl TA, et al. The genetic transformation of *Chlamydia pneumoniae*. mSphere. 2018;3(5). Epub 2018/10/12. doi: 10.1128/mSphere.00412-18 ; PubMed Central PMCID: PMC6180227. 30305318 PMC6180227

[26] Liu Y, Chen C, Gong S, Hou S, Qi M, Liu Q, et al. Transformation of *Chlamydia muridarum* reveals a role for Pgp5 in suppression of plasmid-dependent gene expression. J Bacteriol. 2014;196(5):989–98. Epub 2013/12/24. doi: 10.1128/JB.01161-13 ; PubMed Central PMCID: PMC3957687. 24363344 PMC3957687

[27] Harris SR, Clarke IN, Seth-Smith HMB, Solomon AW, Cutcliffe LT, Marsh P, et al. Whole-genome analysis of diverse *Chlamydia trachomatis* strains identifies phylogenetic relationships masked by current clinical typing. Nature Genetics. 2012;44:413+. doi: 10.1038/ng.2214 22406642 PMC3378690

[28] Jeffrey BM, Suchland RJ, Quinn KL, Davidson JR, Stamm WE, Rockey DD. Genome sequencing of recent clinical *Chlamydia trachomatis* strains identifies loci associated with tissue tropism and regions of apparent recombination. Infection and Immunity. 2010;78(6):2544–53. doi: 10.1128/IAI.01324-09 20308297 PMC2876530

[29] DeMars R, Weinfurter J. Interstrain gene transfer in *Chlamydia trachomatis* in vitro: Mechanism and Significance. Journal of Bacteriology. 2008;190(5):1605–14. doi: 10.1128/JB.01592-07 18083799 PMC2258673

[30] Demars R, Weinfurter J, Guex E, Lin J, Potucek Y. Lateral gene transfer in vitro in the intracellular pathogen *Chlamydia trachomatis*. J Bacteriol. 2007;189(3):991–1003. Epub 2006/11/24. doi: 10.1128/JB.00845-06 ; PubMed Central PMCID: PMC1797294. 17122345 PMC1797294

[31] Suchland RJ, Carrell SJ, Wang Y, Hybiske K, Kim DB, Dimond ZE, et al. Chromosomal recombination targets in *Chlamydia* Interspecies lateral gene transfer. J Bacteriol. 2019;201(23). Epub 2019/09/11. doi: 10.1128/JB.00365-19 ; PubMed Central PMCID: PMC6832074. 31501285 PMC6832074

[32] Suchland RJ, Sandoz KM, Jeffrey BM, Stamm WE, Rockey DD. Horizontal transfer of tetracycline resistance among *Chlamydia* spp. in vitro. Antimicrobial Agents and Chemotherapy. 2009;53(11):4604–11. Epub 2009/08/19. doi: 10.1128/AAC.00477-09 ; PubMed Central PMCID: PMC2772348. 19687238 PMC2772348

