# Supplementary figures and images for: A broad-spectrum cloning vector that exists as both an integrated element and a free plasmid in Chlamydia trachomatis
Source: PLoS One. 2021 Dec 16;16(12):e0261088. doi: 10.1371/journal.pone.0261088 (PMC8675754; doi:10.1371/journal.pone.0261088)

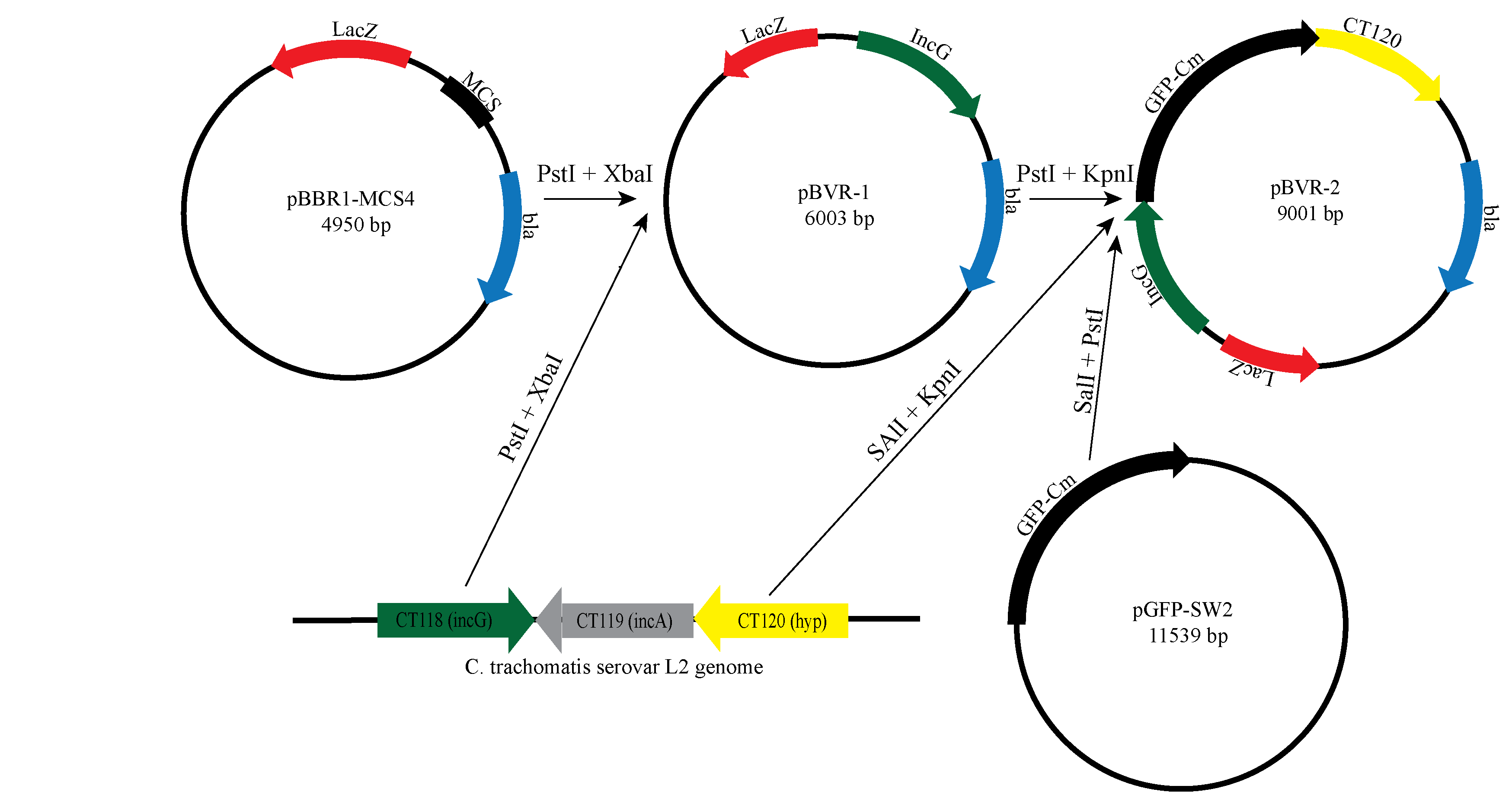

Supplement: S1 Fig — The green fluorescent protein fused with cat is shown in black. (TIF) [file pone.0261088.s001.tif]
