# Supplementary material for: A broad-spectrum cloning vector that exists as both an integrated element and a free plasmid in Chlamydia trachomatis
Source: PLoS One. 2021 Dec 16;16(12):e0261088. doi: 10.1371/journal.pone.0261088 (PMC8675754; doi:10.1371/journal.pone.0261088)
Supplement: S1 Table — (PDF) [file pone.0261088.s002.pdf]

**Table S1. Primers used in this study**			
**Name**	**Sequence (5'-3')**	**Tm**	**Description**
RV238	AACACGCTCTAGAATGCAAATCGAGTCCAGCTAATGAA	57	incG forward with XbaI
RV239	GCAATGCAAAACATAACACCCTG	55	incG reverse
RV240	GCATTCGTCGACGGTTGAGGTACGCGATTGGCTG	60	hyp forward with SalI
RV241	AACATGCTCTAGATATCTGCGCCTGCTTCCCTACATGC	61	hyp reverse with XhoI
RV283	GATGACATGTGATTCGCGTAGG	55	incG Cm:GFp :hyp sequencing forward
RV284	CATTTCACGACAGGCACACC	56	hyp reverse sequencing
RV287	GCATTCGTCGACAGCCCTAGGATCTGGTTTCAGAGAAC	60	Ct incA forward with SalI
RV288	CCATTGCTGCAGTTACTTATCGTCGTCATCCTTGTA-<br>GTCGGAGCTTTTTGTAGAGGGTGATGC	58	Ct incA reverse with PstI and FLAG tag
RV289	CCATTGCTGCAGCTAGGAGCTTTTTGTAGAGGGTGATGC	58	Ct incA reverse with PstI
RV292	TGTAAAACGACGGCCAGTGAGCG	54	M13 forward
RV293	CACACAGGAAACAGCTATGACCATGATTAC	53	M13 reverse
RV294	GTGGTGGATTTGCTCTTGGATTGG	58	upstream region of cloned Ct incG (forward)
RV295	TACTTAGCACCATGTTCTTCCCCTTG	60	downstream of cloned Ct hyp region (reverse)
RV296	CGCTATAAAGACCGTCGGGC	58	GFP reverse to test integration
RV297	GATGGCTTCCATGTCGGCAGAATG	60	GFP forward to test integration
RV305	GCACCAGGTACCGCAGATGCCCGACGG	56	nmP forward with KpnI
RV306	GCATTCGTCGACGTACCCGGGGATCCACC	57	nmP reverse with SalI
RV311	CGACGATTTCCGGCAGTTTCTACAC	59	GFP forward to test integration
RV312	CTGAGCCTCTTTGACCTCTTGCC	60	downstream of cloned Ct hyp region reverse #2
