# Supplementary material for: A broad-spectrum cloning vector that exists as both an integrated element and a free plasmid in Chlamydia trachomatis
Source: PLoS One. 2021 Dec 16;16(12):e0261088. doi: 10.1371/journal.pone.0261088 (PMC8675754; doi:10.1371/journal.pone.0261088)
Supplement: S1 Raw data — (PDF) [file pone.0261088.s003.pdf]

Supplemental Information
Raw gel data for Garvin et al., Figures 3 and 4

1

Fig 4B: Primers 294 and 296

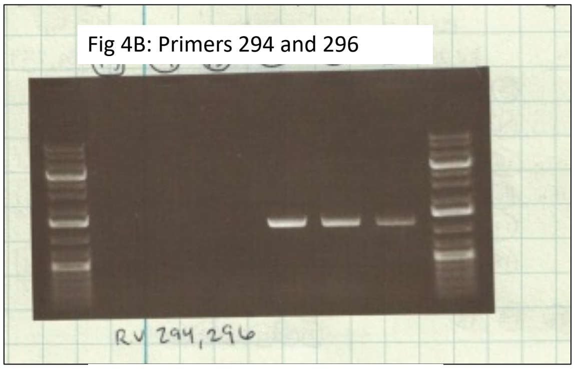

Fig 4B: Primers 240 and 241

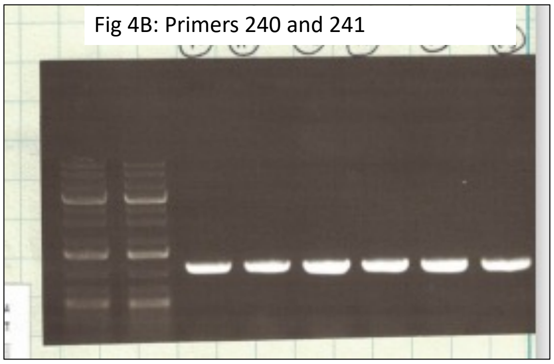

Fig 4B: Primers 295 and 311

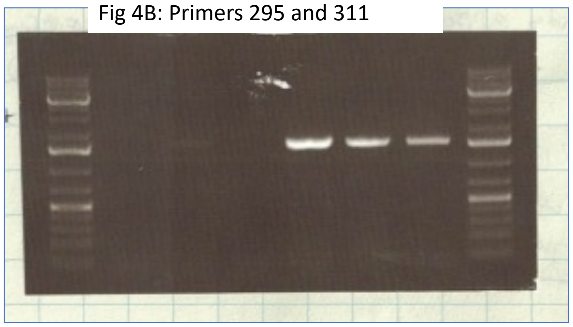

Figure 4. Plasmid pBVR2 integrates into the *C. trachomatis* chromosome at homologous regions. PCR amplification using DNA from the pBVR2 transformants was performed using primers specific for *C. trachomatis* chromosomal DNA, outside of the cloned region (294 or 295), and a primer sequence located within GFP: Cm (296 or 311). Negative controls included non-transformed *C. trachomatis* L2 DNA (**lane 1**), *C. trachomatis* L2 transformed with plasmid pSW2 (**lane 2**), and plasmid pBVR2 DNA alone (**lane 3**). Primers 240 and 241, specific for *ct_120*, were used as a positive PCR control. Lanes (**4-5**) contained chromosomal DNA from the *C. trachomatis* pBVR2 clones 5F and 6B. Lane (**6**) contained chromosomal DNA from the parent *C. trachomatis* pBVR2 transformant prior to cloning, respectively.

Note: all the lanes (1-6) have the same conditions for each gel. The only difference is the primer set used.

2

Molecular mass standards for gels in fig 3 and 4

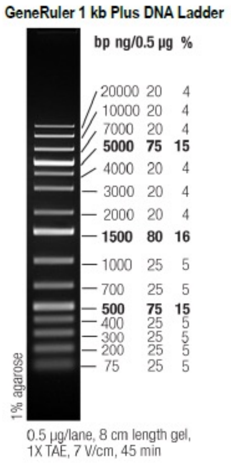

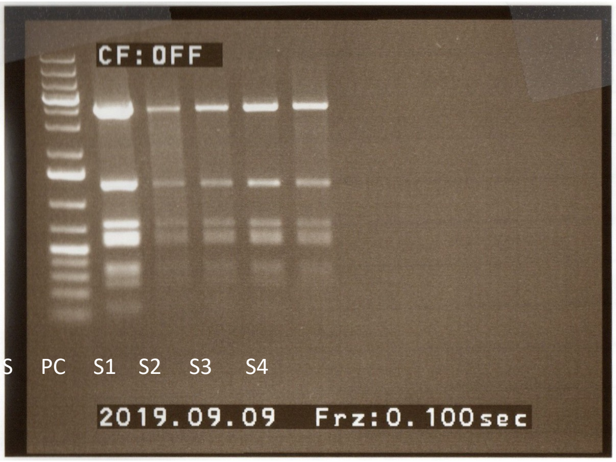

Figure 3. *C. trachomatis* derived pBVR2 plasmid is identical to *E. coli* derived pBVR2 plasmid. Plasmid isolated from *C. trachomatis* and *E. coli* were digested with restriction enzymes *AdeI* and *BglII*. (PC) positive control pBVR2 plasmid raised in dam-/dcm- *E. coli* for initial transformation of *C. trachomatis*. **(S1-S2)** clones of pBVR2 plasmid isolated during round 1 of transformation that was extracted from *C. trachomatis* and transformed into *E. coli* top10 cells. (S3-S4) clones isolated during second round of cloning using same protocol as round 1 clones.

3
